# Determinants of post-traumatic stress disorder among survivors of road traffic accidents in dessie comprehensive specialized hospital North-East Ethiopia

**DOI:** 10.1186/s12888-023-04673-4

**Published:** 2023-03-30

**Authors:** Geleta Mussa Yimer, Yonas Fissha Adem, Yosef Haile

**Affiliations:** 1 Department of Epidemiology and Biostatics’, School of Public Health, College of Medicine and Health Sciences, Wollo University, Dessie, Ethiopia; 2 Department of Public Health, Dessie College of Health Sciences, P.O.Box: 1212, Dessie, Ethiopia; 3 Department of Public Health, College of Medicine and Health Science, Arba Minch University, Arba Minch, Ethiopia

**Keywords:** Post-traumatic stress disorder, Road traffic accident, Determinant factors

## Abstract

**Background:**

Post-traumatic stress disorder is the most common mental disorder occurring among survivors of road traffic accidents. However, it remains under-explored and is not taken into account in the health policies of Ethiopia. Therefore, this study aimed to identify determinant factors of post-traumatic stress disorder among survivors of road traffic accident patients in Dessie Comprehensive Specialized Hospital, North-East Ethiopia.

**Methods:**

A facility-based unmatched case–control study design was employed from February 15 to April 25, 2021, in Dessie Comprehensive Specialized Hospital, with a total sample size of 139 cases and 280 controls selected by using a simple random sampling technique. Data were collected by pretested, interview with a structured questionnaire. The data were entered using Epi-Info, then exported and analyzed using STATA. The bi-variable and multivariable binary logistic regression model was used to identify determinant factors of post-traumatic stress disorder among survivors of road traffic accident. Adjusted odds ratio with a 95% confidence level was used as a measure of association. Variables with a *p*-value less than 0.05 were considered as statistically significant.

**Result:**

A total of 135 cases and 270 controls participated in this study, with a response rate of 97% and 96%, respectively. In the final multivariable analysis, being male [AOR = 0.43, 95% CI: 0.32–0.99], primary educational status [AOR = 3.4, 95% CI: 1.04–11], presence of personal psychiatric history [AOR = 2.12, 95% CI: 1.17–3.92], presence of fracture [AOR = 2.41, 95% CI: 1.2–4.8], witness of death [AOR = 2.25, 95% CI: 1.26–4.30], presence of comorbidity [AOR = 2.29, 95% CI: 1.28–4], good social support [AOR = 0.71, 95% CI: 0.12–0.68] were significantly associated with post-traumatic stress disorder among survivors of road traffic accident patients.

**Conclusion:**

PTSD following road traffic accidents is common. A multi-disciplinary approach was therefore essential in the management of road traffic accident survivors at the orthopedic and trauma clinics. Patients with poor social support, bone fracture, witnessed death, comorbidity, and females should be routinely screened for post-traumatic stress disorder in all road traffic accident survivors.

## Background

Post-traumatic stress disorder (PTSD) is one of the most common mental diseases for traffic accident survivors with serious and long-lasting impacts if untreated [1]. Post-traumatic stress disorder (PTSD) is defined as the persistence of intense, distressing, fearfully avoided reactions to reminders of the accident, alteration of mood, sense of imminent threat, disturbed sleep, and hyper-vigilance for more than one-month duration among traumatic event survivors [2].

The American Psychiatric Association revised the PTSD diagnostic criteria in the Fifth Edition of its Diagnostic and Statistical Manual of Mental Disorders (DSM-5) in 2013. PTSD is newly categorized as a trauma and stressor-related disorder. It was diagnosed by four core symptom clusters: (A) recurrent, involuntary, and intrusive recollections of the event, (B) avoidance of stimuli associated with the trauma, (C) negative alterations in cognitions or moods associated with the event, (D) alterations in arousal and reactivity, including a heightened sensitivity to potential threat lasting more than one-month duration [3].

Those with PTSD are more likely to divorce, have trouble raising their children, have poor partner relations, and find it difficult to strengthen social interaction [4]. It contributes to exacerbating pre-existing mental comorbidities, further leading to lower living standards [5]. Victims are held responsible for long periods of hospitalization and poor recovery in both physical and mental health outcomes [6]. When road traffic accident (RTA) survivors develop PTSD, they are at greater risk of developing other medical diseases such as asthma, hypertension, and peptic ulcer disease [7]. If left untreated, it leads to the individual, as well as community living conditions, becoming more endangered [8].

Globally, PTSD prevalence ranges from 6.3% to 58.3%, and the pooled prevalence of PTSD among road traffic accident (RTA) survivors was 22.25% [9]. PTSD is the leading psychiatric morbidity with magnitude secondary to RTAs, which ranged from 34%, 18%, and 14% at durations of 1, 6, and 12 months respectively [8]. In African studies, rates as low as 8% and as high as 61% reported that RTA victims had developed PTSD [7, 10, 11].

Multiple factors could affect PTSD, including sex and perceiving life-threatening at the time of trauma [12]. Age at trauma, marital status, and educational level were associated with the development of PTSD [13]. Literature showed that the severity of injury (the nature of the accident, the type, and site of injuries) and patients who witnessed death/serious life-threatening injury was proved to develop PTSD [6]. Determinant factors like low income, family history of mental illness, coexisting personal psychiatric illness, and history of prior exposure to trauma were identified as factors of PTSD [14]. A systematic review performed in Europe found that duration of ICU, loss of consciousness, and length of hospital stay were risk factors for PTSD [15]. The duration since the accident, alcohol consumption, and poor social support were other identified determinants of PTSD in RTA survivors [6].

Ethiopia is one of the high-burden RTA countries, with a prevalence of PTSD of 22.8% [16]. In Ethiopia, a considerable effort was done to continuous clinical and mental health professional development, which was increasing. Supportive health education was initiated while caring for orthopedic patients and using different mass media communications in recent years in Ethiopia [17]. Even though those interventions were practiced, there is an increasing rate of RTA, and next to that, survivors are discharged without receiving services such as screening, counseling, and psychological care once they have already developed PTSD.

However, a few studies have been conducted in Ethiopia regarding the prevalence of PTSD among RTA survivors, and there is a lack of data regarding risk factors for PTSD. We hypothesize that PTSD is related to functional disability of the road traffic accident on the victims. Therefore, this study was intended to fill the gaps by assessing the determinant factors of PTSD among survivors of road traffic accident. Since conducting research on PTSD determinant factors is worthwhile for identifying possible risk factors that help to early, appropriate diagnoses and predict possible intervention options [16]. It helps to integrate mental health services in primary healthcare by studying determinant factors of PTSD based on locally available resources [13]. It aids in incorporating PTSD assessment procedures in clinical practices. It also assists in the early identification of populations at risk for PTSD after a patient sustained RTA.

## Method and material

### Study design, setting, and period

A facility-based, unmatched case–control study design was carried out in Dessie Comprehensive Specialized Hospital, which is located in Amhara regional state’s South Wollo zone. DCSH is the primary center for RTA injury admission in the South Wollo zone and neighboring zones and regions (Afar). It serves more than 7 million people. The hospital offers different inpatient and outpatient services (preventive, promotive, curative, and rehabilitative health care services). DCSH serves road traffic accident patients as both outpatients and inpatients service. It has emergency services and general management for RTA patients. Due to its geographical location, DCSH has a high burden of road traffic accident patients and serves for referrals from all woredas of South Wollo zone, Afar regional state, and some areas of Oromia zone. DCSH is working together with Wollo University, to advance orthopedic service through the continuous professional development of surgical nurses, intensive care unit (ICU) nurses, orthopedics, neurosurgeon specialization of general practitioners, and psychiatric professionals. DCSH also has mental health treatment wards for both inpatient and outpatient settings. The study was conducted from February 15 to April 25, 2021.

### Case definitions

Cases: were RTA survivors who had developed PTSD based on post-traumatic stress disorder checklist edition five (PCL-5) score greater than or equal to 40 (PCL-5 ≥ 40).

Controls: RTA patients without PTSD or PCL-5 score less than 40 (PCL-5 score < 40).

### Source population

For cases: All RTA patients with PTSD present at DCSH.

For controls: All RTA patients present at DCSH.

### Study population

For cases; All PTSD patients secondary to RTAs in DCSH during the time of data collection period.

For controls; All RTA patients visited DCSH other than PTSD during the time of data collection period.

### Eligibility criteria

#### Inclusion criteria of cases

RTA survivor patients present at the orthopedic department for clinical care or follow-up evaluation both inpatients and outpatients who were developed PTSD.

#### Inclusion criteria of Controls

Patients present at the orthopedic department both inpatients and outpatients in Dessie Comprehensive Specialized Hospital following RTA who were not developed PTSD.

### Exclusion criteria for both cases and controls

- ✓ RTA patients whose length of duration is less than one month since the accident

- ✓ Patients who had a history of major trauma (war, sexual assault, survivors of disasters) and

- ✓ Patients under the age of 18 years were excluded.

### Study variables

#### Dependent variable

Was post-traumatic stress disorder (PTSD) with their respective codes, Cases = 1 and Controls = 0.

### Independent variables

Was age at trauma, sex, marital status, educational status, occupation status, family history of mental illness, personal psychiatric history, previous RTA, and medical illness. loss of family/ close friend, presence of bone(s) fracture, loss of property, perceived life-threatening events, witnessed death or serious injury, loss of consciousness (LOC), types of injury, duration since RTA, comorbidity, regular income, intensive care unit (ICU) duration, presence of chronic pain, disability, and social support.

### Sample size determination

The sample size calculation was done using Epi Info, statistical software, version 7.2.3.1, by considering all the following assumptions; 2.1 odds ratio, a control to case ratio of 2:1, and 17.88% of controls among the exposed group with social support variable, from previous studies done in Addis Ababa, Ethiopia [16]. There was an accepted error of 5%, a power of 80%, and a 95% confidence level. The sample size obtained considering the above assumptions was 381 (127 for cases and 254 for controls). Adding 10% non-response rate, the total sample size was 419, which is 139 cases and 280 controls.

### Sampling procedure

Among orthopedic patient charts registration, a total of 512 RTA survivors were listed out, then prepared sampled attendants’ sheets, which give a sampling frame, from both inpatients and patients who had been appointed for follow-up services during the data collection period.

Cases and controls were identified based on the screening tool's PCL-5 cutoff point score [3]. All diagnosed cases were selected by counting all cases (interviewed all) when RTA survivors both treated inpatients and took follow-up services in the orthopedic department. All diagnosed cases were included until the required sample size for cases was reached.

Controls were selected using a patient list from prepared sample attendants’ sheets (prepared sampling frame) using registered inpatients and patients who had been appointed for follow-up services during the data collection period. Controls were selected using simple random sampling (SRS) by the lottery method when RTA survivors were both treated as inpatients and received follow-up services in the orthopedic department.

### Operational definitions

Comorbidity: mental health problems with the presence of other psychiatric disorders like depression, and anxiety.

Disability: impairment in social, occupational, or other important areas of functioning [3]. Disability was assessed by the Sheehan disability assessment scale. This was measured with the help of 3 items summed into a single-dimensional measure of global functional impairment that ranges from 3–15. Patients who scored 5 or greater on any of the 3 scales indicated a significant functional disability [18].

Mental disease: a behavioral or mental pattern that causes significant distress or impairment of personal functioning.

Perceived life-threatening: patient feeling his or her life was endangered or believed he or she was dead or faced severe injury during the accident.

PTSD: is characterized by exposure to a traumatic RTA in which the individual witnessed or confronted with RTA which involved actual or threatened death or serious injury or a threat to the physical integrity of self or others and that the individual responded to such an accident with intense fear and/or helplessness or horror. Measured by PCL-5, with responses range 0 to 4, and the total score (range = 0–80). PCL-5 ≥ 40 = PTSD.

RTA: defined as a collision /crash involving at least one road vehicle in motion that could be caused death or injury to people, and destruction of property.

Social Support: assesses perceived availability and satisfaction with support received from family, friends, and other special persons with whom one can share his or her joys and sorrows. Social support was collected by Oslo 3-item social support scale (OSS-3). It is a 3-item questionnaire that provides a brief measure of social support and functioning. In order to score OSS-3, total scores are calculated by adding up the raw scores for each item [1]. The sum of the raw scores has a range from 3–14. A score ranging from 3–8 as poor, 9–11 as moderate, and 12–14 as strong social support [19].

### Data collection tools

Post-traumatic stress disorder is not diagnosed by physical examination or routine laboratory investigation; rather, it is diagnosed using the PCL-5 questionnaire [20]. PCL-5 was a consistent, reliable, and valid tool adopted by DSM-5, which was studied in different populations in different countries over different periods. So it was the gold standard for screening PTSD and was scored to provide a provisional PTSD diagnosis [3].

The post-traumatic stress disorder checklist edition-5 (PCL-5), asks about symptoms in relation to an identified stressful experience. It aims to link symptom endorsements to a specified event; in this study, the event was RTA. It has 20 items, and a 5-point Likert response scale (“0 = not at all,” “1 = a little bit,””2 = moderately," “3 = quite a bit,” "4 = extremely”).

A total symptom severity score (range = 0–80) can be obtained by summing the scores from each of the 20 items that have response options ranging from 0 “not at all” to 4 “extremely”.

The cut point was dependent on the population and the use of the measure [21]. A provisional DSM-5 PTSD diagnosis is obtained from the PCL-5 by considering items rated 2 = ”moderately” or higher as symptoms endorsed following the DSM-5 diagnostic rule [20]. Therefore, according to the DSM-5 rule using instrument tool PCL-5, PTSD is identified at least when the respondent scores 2 or more, i.e. ≥ total sum score of 40 [3].

Social support was assessed by the Oslo 3-item social support scale (OSS-3) [22]. Disability was also screened by the Sheehan disability assessment scale [18].

### Data collection procedures and quality control

The data was collected using structured, interview-administered questionnaires adapted from different works of literature [7, 14, 23, 24]. The questionnaires were translated into Amharic and back to English to ensure consistency. The data was collected by two BSc nurses, who were selected from other health facilities and supervised by one BSc public health professional. Its quality was controlled by designing proper data collection tools, pre-testing, and continuous supervision, and before actual data collection, training was provided to BSC nurse data collectors for two days on the data collection techniques to familiarize them with the tool.

The data collection was conducted in which data collectors contacted study participants while RTA survivors were receiving clinical care and follow-up evaluation services at the orthopedic department. Data quality for PCL-5 scores was checked in the pre-test assessment and gave excellent validity and reliability of the tool (Cronbach’s α = 0.9004). The Oslo 3-item social support scale (OSS-3) measurement was assessed for its reliability and validity in the pre-test of this study, and it was acceptable (Cronbach’s α = 0.6927). The data quality of the Sheehan disability scale was checked for its consistency during the pre-test of this study, and its validity and reliability test was checked, and it was good (Cronbach's α = 0.8515).

### Data analysis and management

Data were entered in Epi-Info, and it was checked, cleaned, and edited before analysis. It was exported to STATA version 14.2.3 for analysis. Frequency distributions and cross-tabulations were used to check for missed values and outliers during the analysis. Descriptive statistics were computed as frequency, percentage, and results were displayed using tables and graphs. The relationship between the independent variable and the outcome variable was determined using a multivariable binary logistic regression model. Model fitness was checked by Hosmer and Lemeshow test, and multicollinearity was checked by VIF. The first bivariable analysis was made for each independent variable to the outcome variable, and those variables resulting in a p-value less than 0.2 were entered into the multivariable binary logistic regression model. In the final model, those variables with a p-value less than 0.05 were considered as statistically significant, and it was presented by odds ratio (OR), with a 95% confidence level (CI) to show the strength and direction of the association.

## Results

A total of 135 cases and 270 controls participated in this study with a response rate of 97% and 96%, respectively.

### Socio-demographic characteristics

Seventy-nine (58.52%) cases and 89 (67.04%) controls were females. The median (IQR) respondents of age in cases were 38(+ 33) years and the median (IQR) ages of controls were 38.5 (+ 28) years. Twenty-one percent of the respondents had less than 1000 Birr monthly income in cases while controls had 17.41% less than 1000 Birr. Of the total respondents, 58(42.96%) among cases and 74(27.41%) among controls were having higher education (diploma and above) respectively. (Table 1.)

**Table 1:** Socio-demographic characteristics of road traffic accident survivors attending DCSH, South Wollo zone, Amhara region, Ethiopia, 2021

Characteristics	Categories	Cases frequency (%)	Controls frequency (%)
**Sex**	Male	56(41.48)	181(32.96)
	Female	79(58.52)	89(67.04)
Age	18–29	42(10.37)	71(17.53)
	30–39	28(6.91)	65(16.05)
	40–49	26(6.42)	41(1012)
	> 50	39(9.63)	93(22.96)
**Education**	No education	5(3.70)	64(23.70)
	Primary(1–8)	44(32.59)	59(21.85)
	Secondary (9–12)	28(20.74)	73(27.04)
	Higher education	58(42.96)	74(27.41)
**Marital status**	Married	86(63.70)	179(66.30)
	Others	49(36.30)	91(33.70)
**Monthly income**	Less than 1000 birr	28(20.74)	47(17.41)
	From 1000 -2999	26(19.26)	78(28.89)
	From 3000–4999	51(37.78)	100(37.04)
	Greater than 5000	30(22.22)	45(16.67)
**Residence**	Urban	81(60.00)	155(57.41)
	Rural	54(40.00)	115(42.59)

**Fig. 1:**
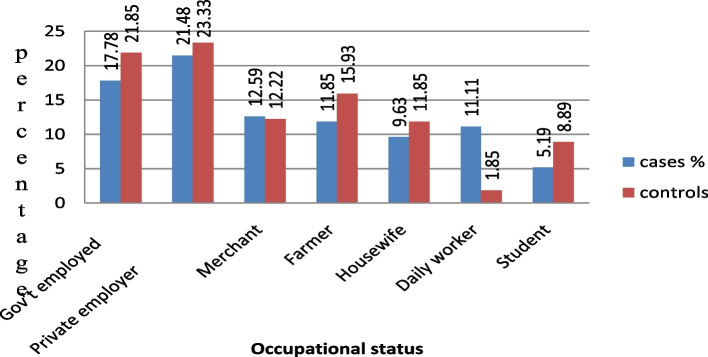
Occupations of participants among RTA orthopedic patients at DCSH, South Wollo zone, Amhara region, Ethiopia, 2021

Among orthopedics RTA patients with PTSD cases and their corresponding controls based on occupational status (Fig. 1).

### Pre-traumatic factors

Among the total participant, 82 (60.74%) cases and 69 (25.56%) controls had a personal psychiatric history. Two third of PTSD cases 81(60.00%) and among controls, 80 (29.63%) were having a family history of mental illness (Table 2).

**Table 2:** Pre-traumatic determinant factors of PTSD among road traffic accident survivors attending DCSH, South Wollo zone, Amhara region, Ethiopia, 2021

Characteristics	Categories	Cases Frequency (%)	Controls Frequency (%)
**Psychiatric history**	Yes	82(60.74)	69(25.56)
	No	53(39.26)	201(74.44)
**Marital status before the accident**	Married	83(61,48)	176(65.19)
	Non married	52(38.52)	94(34.81)
**Family history of mental illness**	Yes	81(60.00)	80(29.63)
	No	54(40.00)	190(70.37)
**Previous RTA**	Yes	76(56.30)	94(34.81)
	No	59(43.70)	176(65.19)

### Peri-traumatic factors

Among survivor orthopedic respondents 104 (77%) of cases and 143 (53%) from total controls suffered from a bone fracture. Study participants from cases 71 (52.59%) and controls 69 (25.56%) experienced a witness of death or serious injury while a road traffic accident occurred.

Eighty-one among 135 cases (60%) respondents reported the death of a family or close friend while the accident happened and only 36% suffered from controls. Among total survivors, 37% in cases and 44% in controls faced compound injury. Patients who had faced lower limp injury both in cases and controls were 19% and 26% respectively (Table 3).

**Table 3:** Peri-traumatic determinant factors of PTSD among road traffic accident survivors attending DCSH, South Wollo zone, Amhara region, Ethiopia, 2021

Characteristics	Categories	Cases Frequency (%)	Controls Frequency (%)
**Bone fracture**	Yes	104(77.04)	143(52.96)
	No	31(22.96)	127(47.04)
**Family lost during accident**	Yes	81(60)	97(35.93)
	No	54(40)	173(64.07)
**Parts of the body injured**	Upper limp	13(9.63)	18(6.67)
	Lower limp	25(18.52)	69(25.56)
	Trunk	10(7.41)	8(2.96)
	Spinal cord	11(8.15)	10(3.70)
	Head	12(8.89)	7(2.59)
	Compound/injury	50(37.04)	120(44.44)
	Not injured	14(10.37)	38(14.07)
**The type of vehicle caused the accident**	Isuzu	20(14.81)	36(1.33)
	Bajaj	28(20.74)	44(16.30)
	Motorcycle	14(10.37)	23(8.52)
	Bus	30(22.22)	77(28.57)
	Mix	43(31.85)	90(33.33)
**Damage of property**	Yes	87(64.44)	163(60.37)
	No	48(35.56)	107(39.63)
**Perceived life threat/endanger**	Yes 96(23.70) No	85(62.96) 50(37.04)	121(44.81) 149(55.19)
**History of LOC**	Yes	70(51.85)	106(39.26)
	No	65(48.15)	164(60.74)

### Post-traumatic factors

Patients with case 67(49.63) developed other comorbid medical diseases whereas 99(36.67) from controls also developed similar comorbidity among traffic accident survivors. Survivors among cases 126(93.33%) developed a functional disability. Unfortunately similar proportion of patients 253(93.70) from the total of controls developed social, occupational, or other important areas of functional impairment secondary to RTA. (Table 4.)

**Table 4:** Post-traumatic determinant factors of PTSD among road traffic accident survivors attending the orthopedic unit of DCSH, South Wollo zone, Amhara region, Ethiopia, 2021

Characteristics	Categories	Cases Frequency (%)	Controls Frequency (%)
**Duration since the accident in months**	1–3	33(24.44)	97(35.93)
	4–5	45(33.33)	59(21.85)
	6–12	48(35.56)	81(30.00)
	> 12 months	9(6.67)	33(12.22)
**Length of hospital stay**	< 1 week	34(25.19)	101(37.41)
	1–2 weeks	40(29.63)	65(24.07)
	3-4 weeks	27(20.00)	64(23.70)
	> 4 weeks	34(25.19)	40(14.81)
**Good health status**	Yes	89(65.93)	117(43.33)
	No	46(34.07)	153(56.67)
**Comorbidity**	Yes	67(49.63)	99(36.67)
	No	68(50.37)	171(63.33)
**New behavior’s**	Yes	70(51.85)	106(39.26)
	No	65(48.15)	164(60.74)
**Regular income**	Yes	74(54.81)	98(36.70)
	No	61(45.19)	172(63.70)
**ICU duration**	Not entering to ICU	73(54.07)	173(64.07)
	Stayed < 6 h	35(25.93)	61(22.59)
	Stayed 7–12 h	22(16.30)	29(10.74)
	Stayed > 12 h	5(3.70)	7(2.59)
**Witness of death**	Yes	71(52.59)	70(25.95)
	No	64(47.41)	200(74.44)
**Functional disability**	Yes	126(93.33)	253(93.70)
	No	9(6.67)	17(6.30)
**Chronic pain**	Yes	109(80.74)	167(61.85)
	No	26(19.26)	103(38.15)

**Fig. 2:**
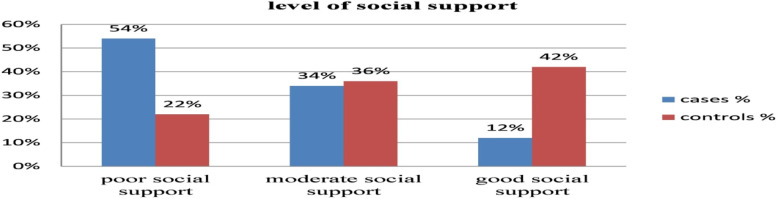
Survivors’ levels of social satisfaction in cases and controls among RTA orthopedic patients at DCSH, South Wollo zone, Amhara region, Ethiopia, 2021

Among orthopedics RTA patients with PTSD cases and their corresponding controls based on the level of social support (Fig. 2).

### Determinants of PTSD among RTA survivors

On the bi-variable, binary logistic regression factors that had a *P*-value ≤ 0.2; include, sex, educational status, marital status, family history of mental illness, psychiatric history, previous RTA, part of body injured, bone fracture, perceived life threatened during the accident, the family lost, current health status, job loss, witnessed serious injury or death of a loved one, ICU duration, long-lasting pain, development of new behaviors, loss of consciousness, regular income after an accident, comorbidity, and social support. These variables were candidates for final multivariable logistic regression analysis (Table 5).

**Table 5:** Bi-variable and multivariable analysis of determinants of post-traumatic stress disorder among RTA survivors attending the orthopedic department of DCSH, South Wollo zone, Amhara region, Ethiopia, 2021

Characteristics	Frequency among Cases (%)	Frequency among Controls (%)	COR(95% CI)	AOR(95% CI)
**Sex**				
Male	56(41.48)	181(32.96)	0.34(0.23,0.53)	0.57(0.3,0.99) *
Female	79( 58.52)	89(67.04)	1	1
**Educational status**				
No education	5(3.70)	64(23.70)	1	3.4(1.04,11)*
Primary(1–8)	44(32.59)	59(21.85)	9.54(3.55,25.7)	2(0.61,6.7)
Secondary	28(20.74)	73(27.04)	4.9(1.79,13.47)	4.9(1.53,15.6)*
* Higher*	58(42.96)	74(27.41)	*10(3.79,26)*	3.4(1.04,11)*
**Family history of mental illness**				
Yes	81(60.00)	80(29.63)	3.56( 2.3, 5.5	1.75(0.95,3.22)
No	54(40.00)	190(70.37)	1	1
**Psychiatric history**				
** Yes**	82(60.74)	69(25.56)	4.5(2.9,7)	2.12(1.17,3.92)**
** No**	53(39.26	201(74.44)	1	1
**Bone fracture**				
** Yes**	104(77.04)	143(52.96)	3 (1.87, 4.75)	2.41(1.2,4.8)***
** No**	31(22.96)	127(47.04)	1	1
**Perceived life threat**				
Yes	85(62.96)	121(44.81)	2.09(1.37,3.2)	1.46(0.80,2.68)
No	50(37.04)	149(55.19)	1	1
**Family lost**				
Yes	81(60.00)	97(35.93)	1.7(1.12,2.59)	0.8(0.43,1.49)
No	54(40.00)	173(64.07)	1	1
**ICU duration**				
Not in ICU	73(54.07)	173(64.07)	1	1
< 6 h	35(25.93)	61(22.59)	1.36(0.83,2.24)	1.39(0.7,2.75)
7–12/hours	22(16.30)	29(10.74)	*1.8(0.97,3.33)*	2.22(0.96,5.14)
12 h	5(3.70)	7(2.59)	1.7(0.52,5.51)	2.26(0.47,10.8)
**Witness of death**				
Yes	7(17.53)	69(25.56)	3.16(2.05,4.89)	2.25(1.26,4)*
No	64(15.80)	201(74.44)	1	1
LOC				
** Yes**	70(51.85)	106(39.26)	1.67(1.1,2.53)	0.7(0.37,1.37)
No	65(48.15)	164(60.74)	1	1
**Comorbidity**				
Yes	67(49.63)	99(36.67)	3.33(2.16,5.14)	2.29(1.28,4)*
No	68(50.37)	171(63.33)	1	1
**Social support**				
Poor	73(54.07)	60(22.22)	1	1
Moderate	46(34.07)	97(35.93)	0.39(0.24,0.64)	0.52(0.28,0.97)*
Good	16(11.85	113(41.85)	0.12(0.06,0.22)	0.29(0.12,0.68)*

*P-value < 0.05, **P-value < 0.01, ***P-value < 0.001.Backward stepwise multiple logistic regression was used to assess the independent effect of explanatory variables

During the multivariable analysis; male sex [AOR = 0.57, 95% CI: 0.3–0.99], primary educational status of participants [AOR = 3.4, 95% CI: 1.04–11], respondents who had higher education and above [AOR = 4.9, 95% CI: 1.53–15.6], personal psychiatric history [AOR = 2.12, 95% CI: 1.17- 3.92], moderate social support [AOR = 0.52, 95% CI: 0.28–0.97], good social support [AOR = 0.29, 95% CI: 0.12–0.68], presence of bone(s) fracture [AOR = 2.41, 95% CI: 1.2–4.8], witnessed of death or serious injury [AOR = 2.25, 95% CI: 1.26–4], presence of comorbid illness [AOR = 2.29, 95% CI: 1.28–4] had showed significant association with the development of PTSD among RTA survivors.

## Discussion

In accordance with this study finding, males had a 43% decreased risk of PTSD as compared to females (AOR = 0.57). This finding was in line with a study done in Addis Ababa Ethiopia, Kenya, and Denmark [14, 23, 24]. This might be due to differences in ways of responding to danger and expressing distress in the two sexes, and females are more likely than males to experience intense fear, horror, or helplessness in response to a traumatic event [25].

The odds of developing PTSD among the participants who had a primary education level or higher education and above were 3.4 and 4.9 times higher as compared to participants who had no formal education. This finding was in line with different studies that were conducted by WHO, and Sweden [26, 27]. As educational status increased more survivors' lives depend on education's benefits than patients with no education. This might be further explained by the survivor's ongoing deterioration of physical and psychological impacts of the accident may be highly affected as the educational status of survivors gets higher [7]. Moreover, the affected victims who knew the probable consequences of the accident were concerned about their personal goals and probable future ambiguities are higher [1].

This study noted that survivor with pre-existing mental diseases was 2.12 times more likely to have PTSD as compared to their counterparts. The current finding was supported by studies conducted in Jimma, southwest Ethiopia; pre-existing untreated depression put two-thirds of individuals at risk of PTSD when exposed to a road traffic accident [28]. The current finding was also supported by international studies from Kenya, Taiwan, and Germany [29–31]. This could be due to having another psychiatric disease increasing the risk of developing PTSD, which leads to poor long-term health outcomes, including impaired physical functioning and a lower self-reported quality of life [32].

The other independent predictor that had a very strong association with PTSD was a bone fracture. Study participants who had experienced a bone fracture were 2.41 times more likely to have PTSD as compared to those who did not have a bone fracture. This result was supported by the study done in Kenya [33]. Moreover, RTA patients who had a bone fracture were highly likely to develop a disability, and psychosocial problems [34]. Those effects might cause a double burden of public health problems for survivors, which further increases the risk of PTSD. This finding was also supported by a study done on survivors who had suffered bone fractures, had undergone major surgical operations like amputation, and were more likely to be at risk than patients without bone fractures [35]. This might be due to the survivor's long-time hospitalization, inability to return to their previous job, persistent absence from previous business activity, and inability to coordinate family integration, which was mainly due to survivors’ experiences with a bone fracture [36].

The odds of witnessing death or a serious life-threatening injury were 2.25 times more likely to develop PTSD as compared to their counterparts. Likewise, a study conducted in Bahir Dar, Ethiopia, showed that witnessing death or serious life-threatening injury during an accident was significantly associated with PTSD [7]. This is also reported in WHO mental PTSD survey [37]. The reason could be the repeated, disturbing memories; thoughts of stressful experiences did have an association with PTSD. Another possible explanation could be those clients with this imagination might lead to survivor's attack on the accident happening again and again [14].

The odds of having a co-morbid psychiatric disorder were 2.29 times more likely to have PTSD as compared to those who had no co-morbid psychiatric disorder. This is consistent with studies conducted in Ethiopia [16]. A similar finding was noted in the USA, major depression disorder (MDD) was the first comorbid that worsen the development of traffic victims to PTSD [27]. This might be due to the reason that patients who have co-morbid psychiatric disorders were increasing their risk to develop PTSD. Survivors suffering from co-morbid psychiatric disorders have poor long-term health outcomes, including impaired physical functioning and a lower quality of life [7].

Social support was significantly associated with PTSD, in which those participants with moderate social support and strong social support were 0.48 and 0.71 times less likely to have PTSD as compared to those with poor social support. This finding was supported in studies done in different countries [38, 39]. This might be because a lack of social support after exposure to traumatic injury may lead to poor mental health since positive social support appears to mitigate the negative effects of traumatic injury, and those who have poor social support may not develop proper coping strategies after trauma [36].

### Strength and limitation of the study

The study assessed only the latest RTA subjects, being treated for. Other different traumas that could have occurred in the subjects’ lifetimes were not taken into account. These could have an influence on these results. However, its major strength was used a standardized instrument for measuring PTSD.

## Conclusions

A road traffic accident was one of the traumatic experiences that caused PTSD. Patients with poor social support, bone fracture, witnessed death, comorbidity, and females should be routinely screened for post-traumatic stress disorder in all road traffic accident survivors. Orthopedic clinics should develop guidelines to screen for and treat PTSD among survivors of RTA.

## Data Availability

The datasets generated and analyzed during the current study are not publicly available due to the risks in identifying participants as true anonymization would be difficult to guarantee, but subsets of the data can be available from the corresponding author upon a reasonable request.

## Abbreviations

DCSH: Dessie comprehensive specialized hospital
DSM-5: Diagnostic and statistical manual of mental disorders
ICU: Intensive care unit
LOC: Loss of consciousness
RTA: Road traffic accident
PTSD: Post-traumatic stress disorder
WHO: World health organization
